# Simulation of bonding effects in HRTEM images of light element materials

**DOI:** 10.3762/bjnano.2.45

**Published:** 2011-07-19

**Authors:** Simon Kurasch, Jannik C Meyer, Daniela Künzel, Axel Groß, Ute Kaiser

**Affiliations:** 1 Central Facility for Electron Microscopy, Group of Electron Microscopy of Materials Science, University of Ulm, 89081 Ulm, Germany; 2 New Address: University of Vienna, Department of Physics, 1090 Vienna, Austria; 3 Institute of Theoretical Chemistry, University of Ulm, 89069 Ulm, Germany

**Keywords:** chemical bonding, DFT, graphene, HRTEM

## Abstract

The accuracy of multislice high-resolution transmission electron microscopy (HRTEM) simulation can be improved by calculating the scattering potential using density functional theory (DFT) [1–2]. This approach accounts for the fact that electrons in the specimen are redistributed according to their local chemical environment. This influences the scattering process and alters the absolute and relative contrast in the final image. For light element materials with well defined geometry, such as graphene and hexagonal boron nitride monolayers, the DFT based simulation scheme turned out to be necessary to prevent misinterpretation of weak signals, such as the identification of nitrogen substitutions in a graphene network. Furthermore, this implies that the HRTEM image does not only contain structural information (atom positions and atomic numbers). Instead, information on the electron charge distribution can be gained in addition.

In order to produce meaningful results, the new input parameters need to be chosen carefully. Here we present details of the simulation process and discuss the influence of the main parameters on the final result. Furthermore we apply the simulation scheme to three model systems: A single atom boron and a single atom oxygen substitution in graphene and an oxygen adatom on graphene.

## Introduction

Conventional HRTEM image simulation so far neglects all kinds of interatomic interactions within the specimen by calculating the total specimen potential as a superposition of isolated atom potentials [3]. It is generally known that the state of an atom is, of course, influenced by its environment and hence techniques that are more sensitive to changes in the electronic state, such as electron energy loss spectroscopy [4] or scanning tunneling microscopy [5], make use of advanced simulation methods to model the specimen.

In 1997, Gemming and Möbus performed ab-initio HRTEM simulations of ionic crystals and justified the use of conventional image simulation [1]. About ten years later, and after enormous improvement in electron optics and the resolution of the TEM by means of aberration correction [6–7], Deng et al. [2,8] performed DFT based HRTEM calculations for bulk oxides and found that chemical bonding should be detectable and in practice is hindered only by the poor specimen quality obtained by ion-beam thinning. Furthermore they pointed out that it is possible to study charge transfer by other techniques such as convergent beam electron diffraction [9–10] but all methods available can only offer global information as they observe the charge distribution in reciprocal space. In contrast, the observation of the same effect in real space using HRTEM would result in local information, which would open new frontiers for electron microscopy [2,11].

**Figure 1:**
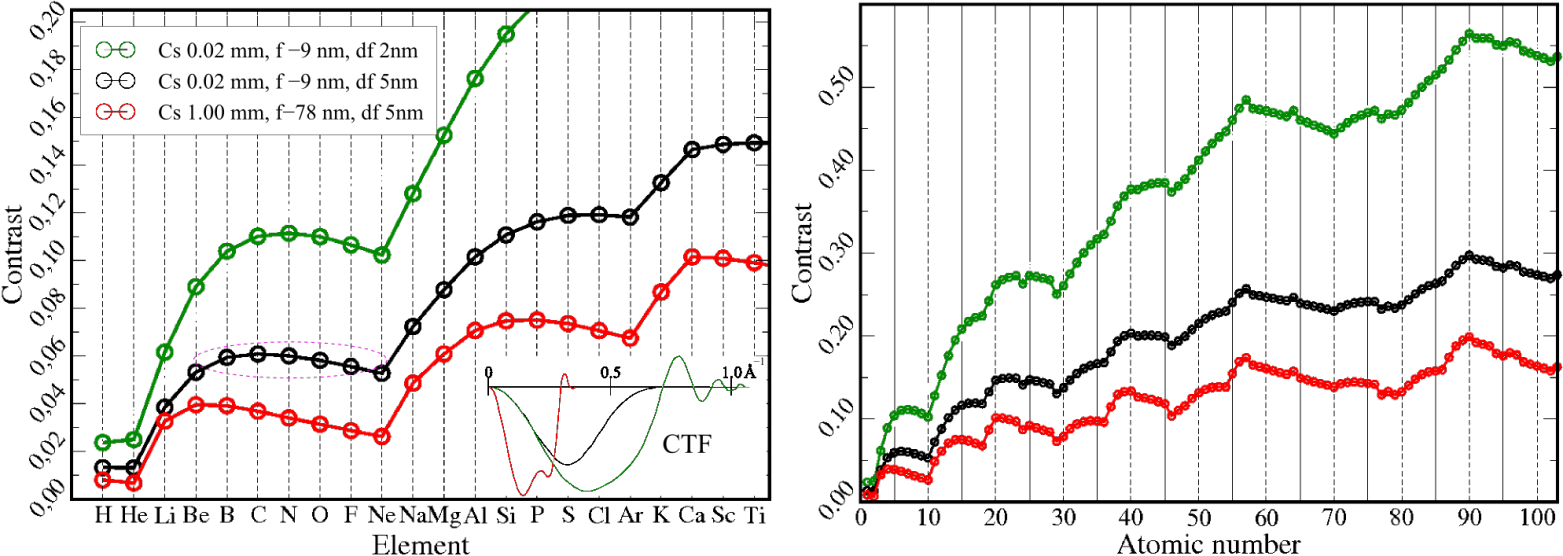
Analysis of atomic contrast for different TEM conditions at 80 kV obtained using a code of E. Kirkland [4]. The red curve corresponds to an uncorrected microscope and the black one to a state of the art *C*_s_ corrected microscope. The green curve is obtained when, in addition to *C*_s_ correction, the focus spread is reduced. This results in better resolution and can be achieved by a monochromator or a *C*_s_ corrector (inelastic scattering is neglected). The phase contrast transfer functions for the different microscopes and corresponding values of spherical aberration *C*_s_, defocus *f* and focus spread *df* are shown in the inset. Importantly, one can find combinations of elements (such as B, C, N, O, marked by the dashed circle), where the neutral-atom contrast differences are very small and hence may potentially be dominated by bonding effects.

Previous studies were focused on bulk oxides, because they are known to have strong ionic bonds. Our target materials, in contrast, are two dimensional crystals such as graphene and hexagonal boron nitride as they offer an outstanding specimen quality that has not been achieved for bulk materials so far: Their thickness is perfectly defined (one atomic layer) and it is possible to find areas without defects and without amorphous top and bottom layers. Furthermore both of our target materials are built from exclusively light elements where strong bonding effects can be expected because most of their electrons are valence electrons. Another important factor for the experimental detection of these effects in HRTEM is that the contrast of boron, carbon, nitrogen and oxygen is almost identical under our imaging conditions [12] (shown by the black curve in Figure 1). Hence even small contrast variations are relatively easy to detect.

Due to these improvements in specimen quality, for the first time, we were able to measure the influence of charge redistribution on the HRTEM image contrast experimentally for two different materials, namely nitrogen doped graphene and single-layer hexagonal boron nitride [13]. This result has two important implications: First, chemical bonding gives small corrections to the atomic contrast in the TEM, which has to be kept in mind whenever weak signals are analyzed. Second, and probably more importantly, the HRTEM image is not only governed by structural information but also contains information about the electronic state of the specimen. This allows the study of the electron charge distribution in point defects and other nanoscaled objects that can not be accessed by diffraction experiments.

Here we give detailed information on the DFT based simulation used in [13] and explain the analysis for three model systems: A single atom boron and a single atom oxygen substitution in graphene and an oxygen adatom on graphene.

## Experimental

### Modeling the HRTEM image formation

High resolution TEM image simulation can be separated in three main parts: First the interaction between the incident electron wave and the specimen is modeled and the specimen exit wave is obtained. Afterwards the specimen exit wave becomes the ”object” for the imaging system of the electron microscope, which produces the image intensity impinging on the recording medium [14]. Finally, the characteristics of the detector are taken into account [15].

The interaction with the specimen is described by a very simple scattering process where the incident high energy electron is scattered by the combined Coulomb potential of all atomic nuclei and electrons within the specimen. Mathematically one has to solve a relativistic version of the Schrödinger Equation 1, where 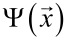 is the wave function of the electron at position 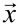, *m* is the relativistic mass of the electron and 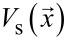 is the specimen potential.

[1]
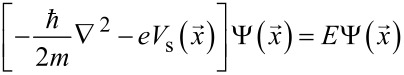


In the limit of high energy electrons, backscattering can be neglected and Equation 1 can be solved using the multislice algorithm. In this study we focus on single layer materials of light elements. Hence the exit wave can be calculated (in a single-slice approximation) by Equation 2, where σ is the interaction parameter and *V*_z_ is the projected specimen potential [3]. In addition, for these structures and our imaging conditions, it turned out that the linear image approximation (Equation 3) is justified, as found by comparison of the result with the standard calculation. The amplitude spectrum of the wave in the imaging plane ψ_image_(*q*_x_, *q*_y_) can be derived from the Fourier space specimen exit wave ψ_ex_(*q*_x_, *q*_y_) by multiplication with the objective lens phase factor function exp [*i*χ(*q*_x_, *q*_y_)], where χ(*q*_x_, *q*_y_) depends on the defocus Δ*f*, spherical aberration *C*_s_ and higher order aberrations [14]. The exact expression of χ(*q*_x_, *q*_y_) can be found in [16]. Because the structures studied here are weak scatterers, the linear imaging condition is justified and, for an incident plane wave, the final image intensity is given by Equation 4 [14].

[2]
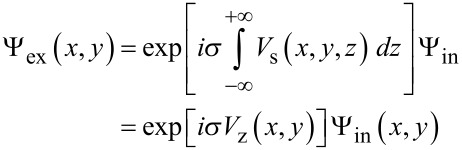


[3]
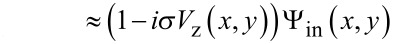


[5]
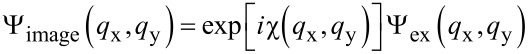


[4]
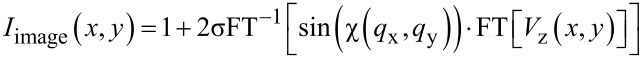


Of course this is a very simple model of the real scattering process, which neglects all kinds of inelastic processes by assuming that the state of the specimen is not at all influenced by the presence of the high energy electrons. Nevertheless, it is well established for HRTEM simulation and in this work we use exactly the same framework (with all its limitations) but focus on a very fundamental question: How do we obtain the scattering potential? The standard approach is to calculate the total specimen potential as a superposition of isolated atom potentials, which have been calculated previously for each element by solving the quantum many body problem for all electrons and the nuclei of a single atom. One example are the potentials published by Doyle and Turner in 1968 [17]. Their paper was based on atomic potentials obtained by relativistic Hartree–Fock self-consistent field calculations performed by Coulthard in 1967 [18], where the main assumption was that the atomic charge distribution is spherically symmetric.

A more accurate way is to include electronic interactions between atoms in the specimen by DFT. In this way, ionic atoms with non-spherical electron distributions can be modeled without any a priori knowledge.

### How to obtain DFT potentials

The DFT calculation was performed in two steps: First we performed a structure optimization of an initial atomic configuration by using the very fast and efficient pseudopotential DFT code VASP [19]. Unfortunately it was not possible to extract the total electrostatic potential directly from the pseudopotential calculation as it only offers the self consistent valence charge density but the total charge density is needed. Hence, in a second step, we used the relaxed structure to set up an all electron DFT calculation, and therefore we used the WIEN2k [20] DFT software. Furthermore, WIEN2k has the significant advantage that, besides offering access to the total electron charge density and corresponding X-ray scattering factors, in addition, the calculation of the total Coulomb potential (including all electrons and nuclei within the unit cell) is already implemented. Deng and Marks [8] used the X-ray scattering factors, while our method directly makes use of the available potential file.

**Figure 2:**
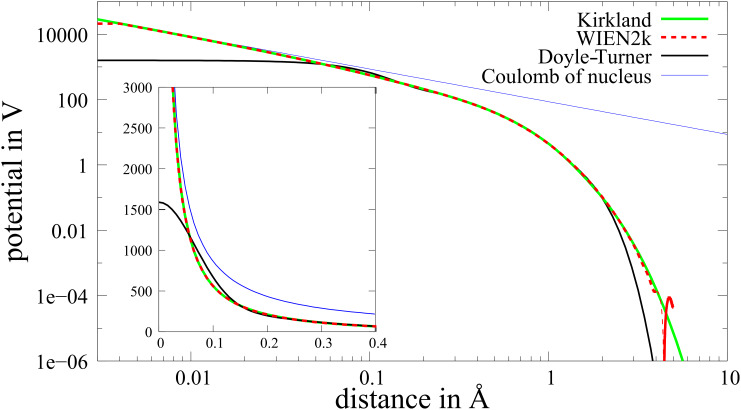
WIEN2k starting potential (red) compared to Doyle–Turner (black) and Kirkland (green) potentials. The blue line corresponds to the unscreened Coulomb potential of a carbon core. The WIEN2k potential linescan has a resolution of 250 ppÅ and was normalized in such a way that the smallest value is equal to zero. The WIEN2k and Kirkland potential show very good agreement.

A very important cross-check is to compare the WIEN2k potential to other potentials used in HRTEM simulations. This is easy to achieve, because the starting point for the DFT calculation (before the first iteration cycle) is also built up from isolated atom potentials, and the subsequent iteration process, searching for a self-consistent field (SCF) solution, acts as a minor perturbation to the initial potential. In Figure 2 we compare the initial WIEN2k potential of a single carbon atom to Kirkland [3] and Doyle–Turner [17] isolated atom potentials. The WIEN2k potential was obtained by putting a single carbon atom into a 10 Å × 10 Å × 10 Å unit cell and calculating a linescan of the electrostatic potential with a resolution of 250 points per Angstrom (ppÅ). Far from the core, the WIEN2k potential approaches a non-zero constant value. In order to obtain the usual normalization, the potential was shifted (i.e., smallest value was set to zero).

The 3D unit-cell potential is stored in the file case.vcoul and WIEN2k comes with utility software (lapw5 and lapw5c) to extract linescans and 2D slices from this file. Hence a point grid of the 3D potential can be extracted by combining subsequently calculated 2D slices using the wien2venus script written by Masao Arai [21]. Difficulties arise from the fact that the potential is divergent near the positions of the atomic nuclei but equidistant discretization is performed. Furthermore the total number of sampling points is rather limited due to limited computer time. Usually this sampling problem is overcome by smoothing the analytical 3D potential before the discretization is performed and, in this way, a much smaller sampling rate can be used (typically 10 ppÅ). In Figure 2 this smoothing can be seen very well in the case of the Doyle–Turner potential, which is not divergent near the nucleus. However, in practice this was not possible here because we can only access the WIEN2k potential via the utility software.

**Figure 3:**
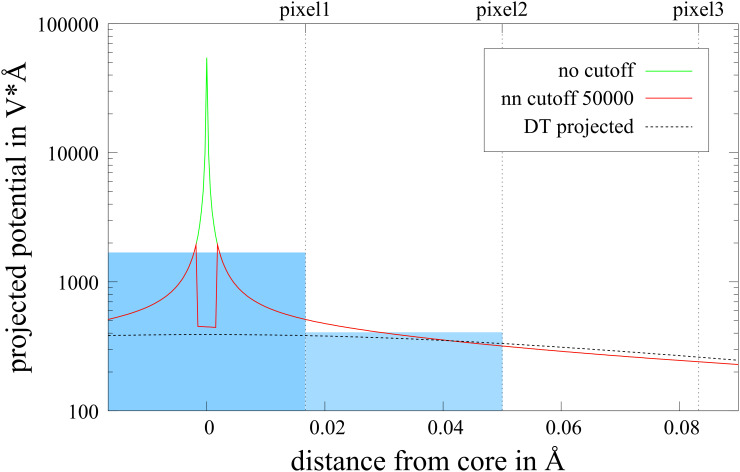
Projected potential near the core at very high resolution (green line). The blue boxes indicate the mean value of the green line at a resolution of 30 ppÅ. That means that the top value of these boxes corresponds to the ”true” projected potential of the corresponding volume element. The red line is obtained using nearest neighbor cutoff. For comparison, the projected Doyle–Turner potential is plotted as a dashed black line.

We analyzed the sampling error in more detail by comparing the dependence of the projected potential from the position of the sampling point at a constant sampling rate of 30 ppÅ in the *z*-direction (parallel to the incident beam), which turned out to be a realistic compromise, but a much higher rate in the perpendicular direction. After projection along the *z*-direction, this results in the projected potential printed in green in Figure 3. From this it is possible to study the error that is made when this function is discretized using a smaller number of sampling points.

The blue boxes in Figure 3 indicate the mean value within one pixel at a resolution of 30 ppÅ, which corresponds to the ideal value within this quadrant. Hence the deviation from the top of the blue box indicates the sampling error. Interestingly, for 30 ppÅ, the sampling error is significant only for the center pixel and is caused by a single value in the 3D potential. We use a very simple method to handle this problem: Whenever a value in the 3D potential is higher than some cutoff value, we change this value to the highest value in the neighboring pixels. In this way the obtained projected potential value of the central pixel is in the range of the ideal value within a factor of three, instead of being off by up to two orders of magnitude (compare red and green curves in Figure 3). This very crude approach can be used, because the fraction of the intensity that interacts with this part of the potential remains negligibly small.

In order to be more flexible, we modified the wien2venus script: First, we included the possibility to shift the slicing volume with respect to the DFT unit cell. In this way it is possible to avoid sampling points very close to the nuclei. Second, it is now possible to slice sub-volumes. This can be used to speed up the calculation, because several sub-volumes can be sliced at the same time and vacuum regions can be skipped. The modified version of the script can be found in the Supporting Information (Supporting Information File 1).

Once an accurate 3D potential is obtained and renormalized it can be used for TEM image simulation. Thereby each direction of the incident beam can be modeled by rotating the 3D potential using linear interpolation algorithms.

### Influence of DFT parameters

In order to set up meaningful DFT calculations, it is always necessary to do convergence tests of the main parameters such as k-points and basis set size [22]. Usually the convergence is tested with respect to the total energy and the electric field gradients. This was done using ideal graphene as a test structure. Interestingly, we find that the main quantity that we are interested in, i.e., the projected electrostatic potential, is not very sensitive to the DFT input parameters: The absolute differences between the DFT and IAM potentials are in the range of 10–30% where the influence of the DFT parameters is smaller than 1.5% (for details see Supporting Information File 2).

## Example calculation

As we expect bonding effects to be strongest in exclusively light element materials, we applied this simulation scheme to different types of defects in graphene. The single atom substitutions, where one carbon position is occupied by another atomic species, turned out to be the ones that can be most easily accessed experimentally because the graphene structure remains almost undisturbed. Hence, bonding effects can easily be separated from structural changes by analyzing the deviations from the regular lattice contrast. For vacancies and adatoms the contrast analysis is much more difficult, due to changes in both, structural and electronic configuration. Nevertheless, the influence of chemical bonding on the final TEM image can be detected for all of them.

### Boron and oxygen substitution in graphene

**Figure 4:**
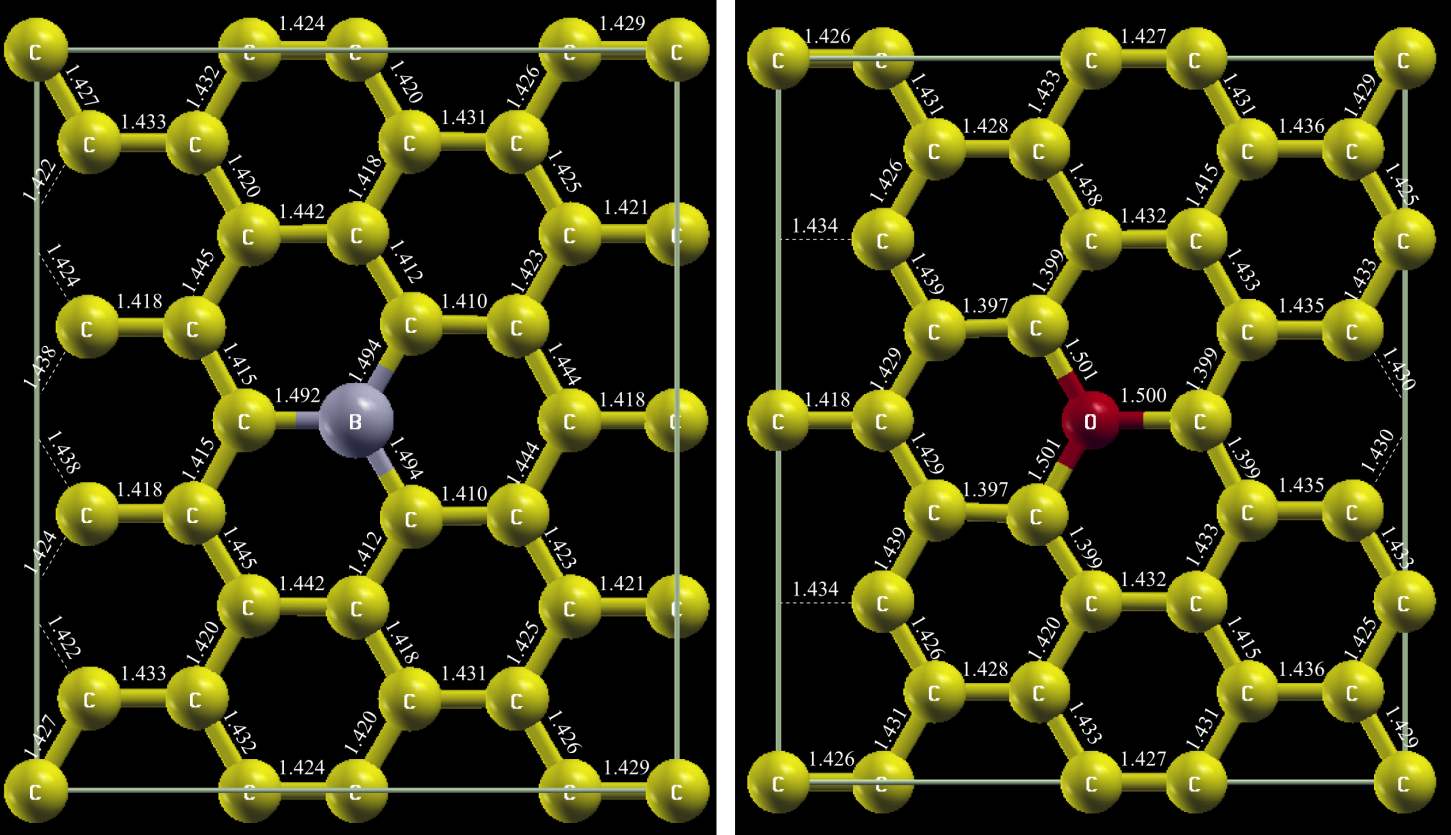
Relaxed structure model of boron and oxygen substitution in graphene. Bond lengths are given in Å.

The structure models obtained from the VASP relaxation are shown in Figure 4. Details on the relaxation process can be found in the supplementary information of [13].

The WIEN2k calculation for the boron substitution was performed using the generalized gradient approximation (GGA) for the description of the exchange-correlation effects [23] with the following set of technical parameters: Separation energy −5.5 Ry, 6 × 6 × 1 k-points, RKMAX = 7 and GMAX = 12. For the calculations including oxygen atoms, the parameters were modified to: −6 Ry, 4 × 4 × 1 k-points, RKMAX = 8 and GMAX = 16. Both calculations were performed in a spin-polarized fashion, and the linearization energies were set automatically.

The effect of charge redistribution due to chemical bonding can be studied by comparing the initial charge density (before the first iteration cycle, labeled IAM) and the self-consistent charge density after the WIEN2k calculation has converged (labeled DFT). The same is done for the potentials. This approach has the advantage that subsequent processing steps, such as the TEM image simulation, influence the quantities obtained by IAM and DFT in exactly the same fashion. The only difference is that the latter includes chemical bonding while the former does not.

The 3D potentials were sliced with a resolution of 30 ppÅ, normalized and projected along the *z*-direction using a cutoff of −50 kV, as described above. The same was done for the all-electron charge density (stored in the file clmsum) where the renormalization and the cutoff was skipped.

**Figure 5:**
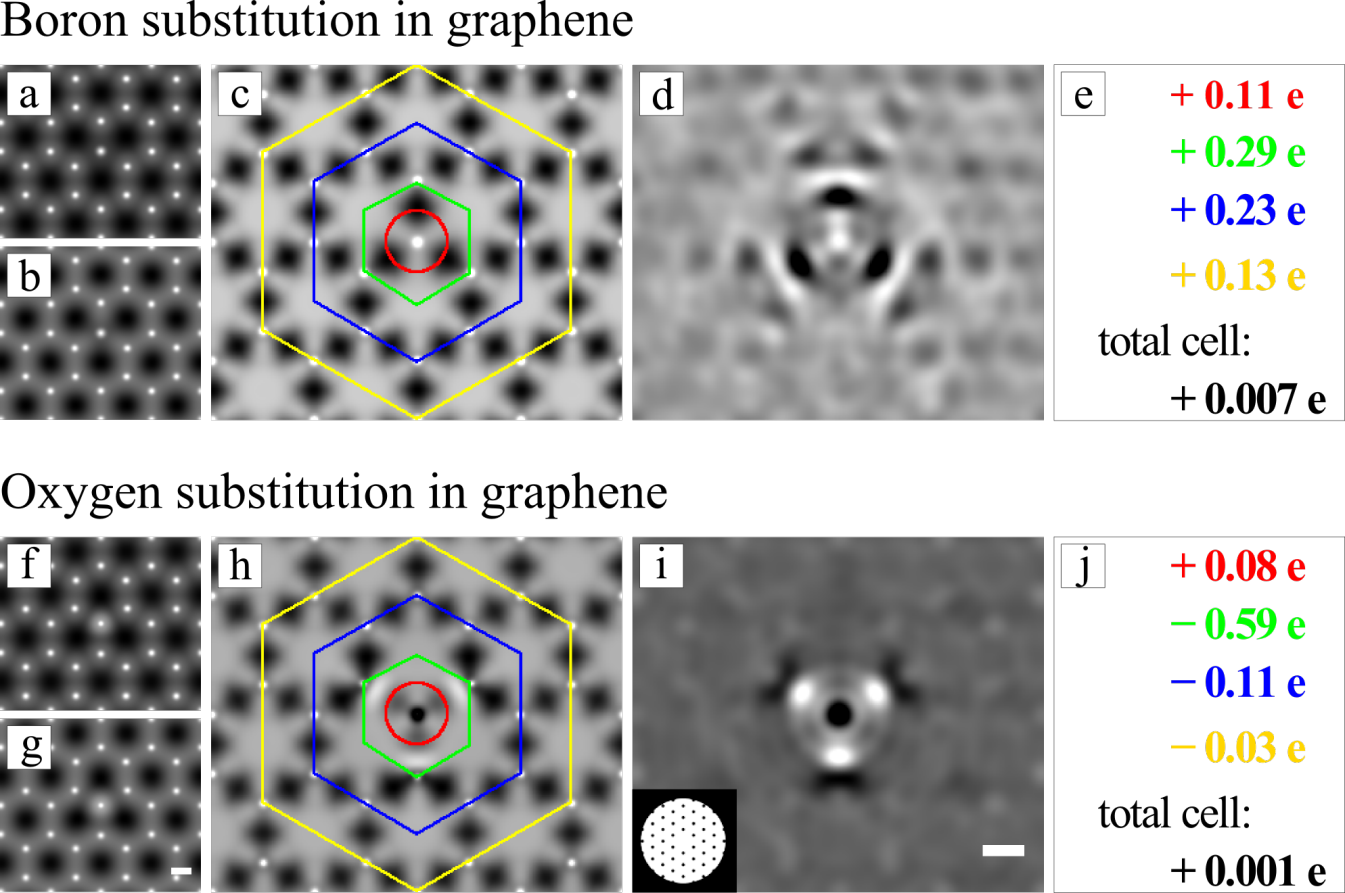
Analysis of the projected electron charge density of the boron (top) and oxygen substitution in graphene (bottom line). a) and b) show the projected charge density of the boron substitution for the neutral and bonded configuration respectively. c) shows the absolute difference between a) and b) where dark contrast corresponds to an increased electron density in the bonded configuration. This difference is integrated over the indicated areas and the values are given in e), where positive values correspond to an increased number of electrons in this area due to bonding. The diameter of the red ring is exactly half of the distance between the substitution and the neighboring carbon atoms and the green hexagon is exactly at the position of the neighboring carbons. The changes in the electron charge density, introduced by the substitution atom can be seen best in d), which is obtained after the periodic component of c) is removed by a Fourier filter, which is shown as inset in i). f)–j) show exactly the same analysis for the oxygen substitution. Scale bars are 1 Å.

In Figure 5 we analyze the difference in the charge density. In the difference images (panel c and h) the sp^2^ hybridization of the graphene lattice is clearly visible by the dark contrast between the carbon atoms meaning that the charge density of the bonded configuration is increased in this area. Interestingly, for the boron as well as for the oxygen substitution we find big differences in the charge density at the three neighboring carbon atoms, whereas the substitution atom itself remains almost neutral. After removing the periodic signal from the difference images, using a Fourier filter (panel d and i), a dipole shaped rearrangement of the electrons at the carbon atoms next to the substitution is detected. Comparing the boron and oxygen case we find that the polarization of the carbon atoms is almost exactly opposite: The electron density in the area surrounding the boron atom (green hexagon) is increased, while for the oxygen atom it is decreased. This should result in a decrease of the boron potential due to stronger shielding of the core potential and reduced contrast in the TEM image. For oxygen, on the other hand, we expect to have a stronger signal in the TEM image due to weaker shielding.

**Figure 6:**
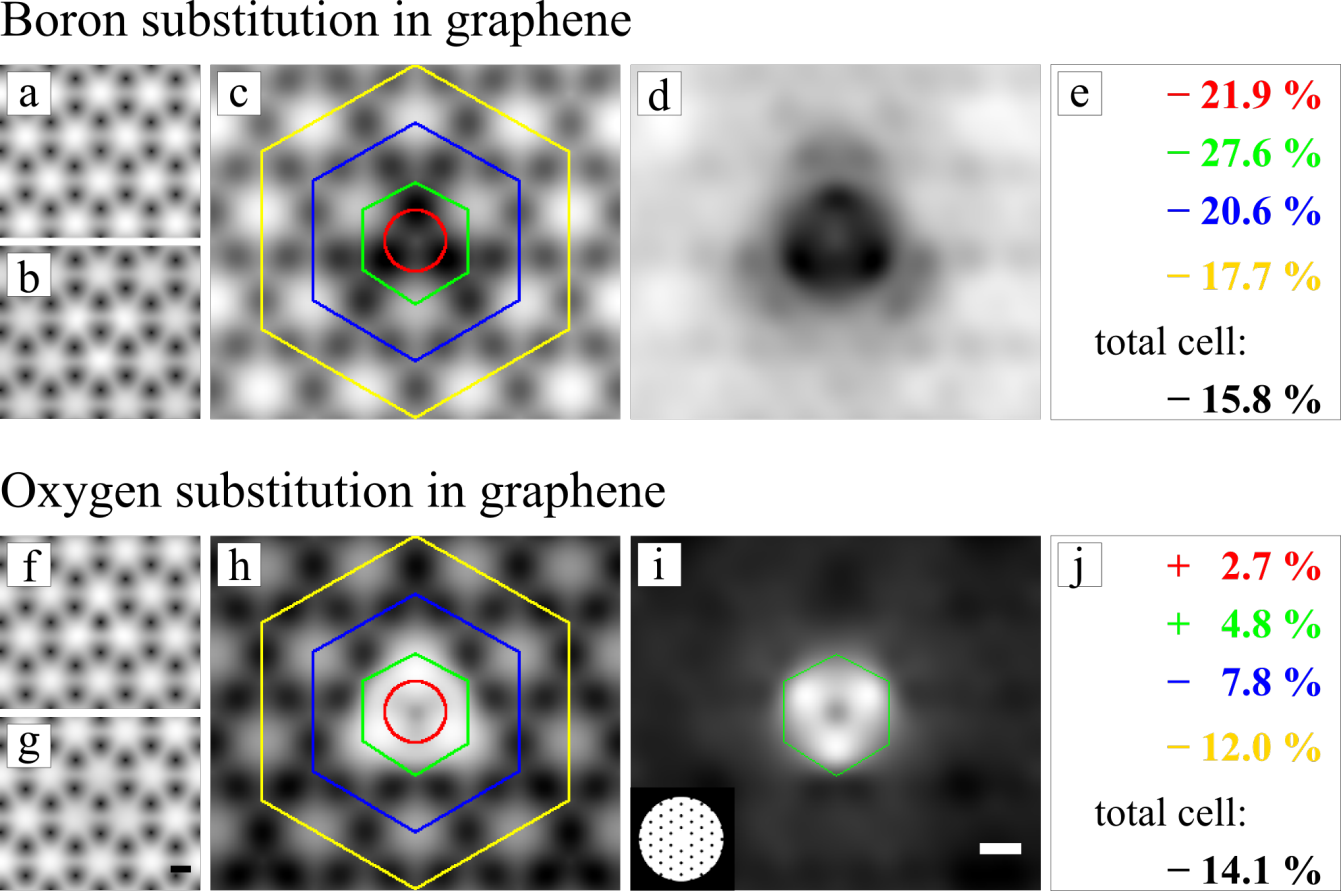
Analysis of the projected potential of the boron (top) and oxygen substitution in graphene (bottom line). a) and b) show the projected potential of IAM and DFT in logarithmic scaling, where the absolute difference image c) is displayed on a linear greyscale. Dark contrast in c) and d) corresponds to a decrease in the projected potential in the bonded configuration due to stronger shielding of the nuclear potential by electrons. The changes of the potential inside the marked areas in c) are given in e). Because the total potential is changing it is easier to compare relative differences. f)–j) show exactly the same analysis for the oxygen substitution. Scale bars are 1 Å.

This is exactly what we find when analyzing the projected potentials. Dark contrast in the filtered difference images in Figure 6 corresponds to a decreased projected potential in the bonded configuration. The increase of the oxygen and the decrease of the boron potential (compared to the IAM) is clearly visible. Besides these obvious differences two more subtle conclusions can be drawn from the potential analysis. First, we find that, also for the potential, the difference is not sharply located at the position of the substitution atom but instead spreads over further atomic distances. This results in low frequency information about the defect. The transfer of this information can be enhanced in the TEM by working at higher defocus. Second, for both cases the total potential is decreased by about 15% (see total change in panel e and j of Figure 6). This change in the mean inner potential is well known and was previously studied for semi-conducting materials by Schowalter et al. [24]. For ideal graphene we find a difference in the mean inner potential of 15.5%. Interestingly this results in an overall loss of contrast in the final TEM image of approximately 8%. However, this is only a minor contribution to the Stobbs factor [25], which is in the range of 50–80% and is used to fit simulated and experimental TEM image intensities.

After analyzing the DFT results we now want to study how the charge redistribution influences the observed contrast in the final TEM image, which is obtained by applying Equation 4. This calculation was performed for two different values of defocus: Scherzer defocus *f*_1_ = −9 nm and *f*_2_ = −18 nm, where the graphene lattice reflection is in the second extremum of the CTF. The former is the standard condition for high resolution TEM, whereas the latter offers better transfer of low spatial frequencies resulting in enhanced contrast of the substitution defects. The other parameters were: High tension 80 kV and spherical aberration *C*_s_ = 0.02 mm. Higher order aberrations were not taken into account in this study.

**Figure 7:**
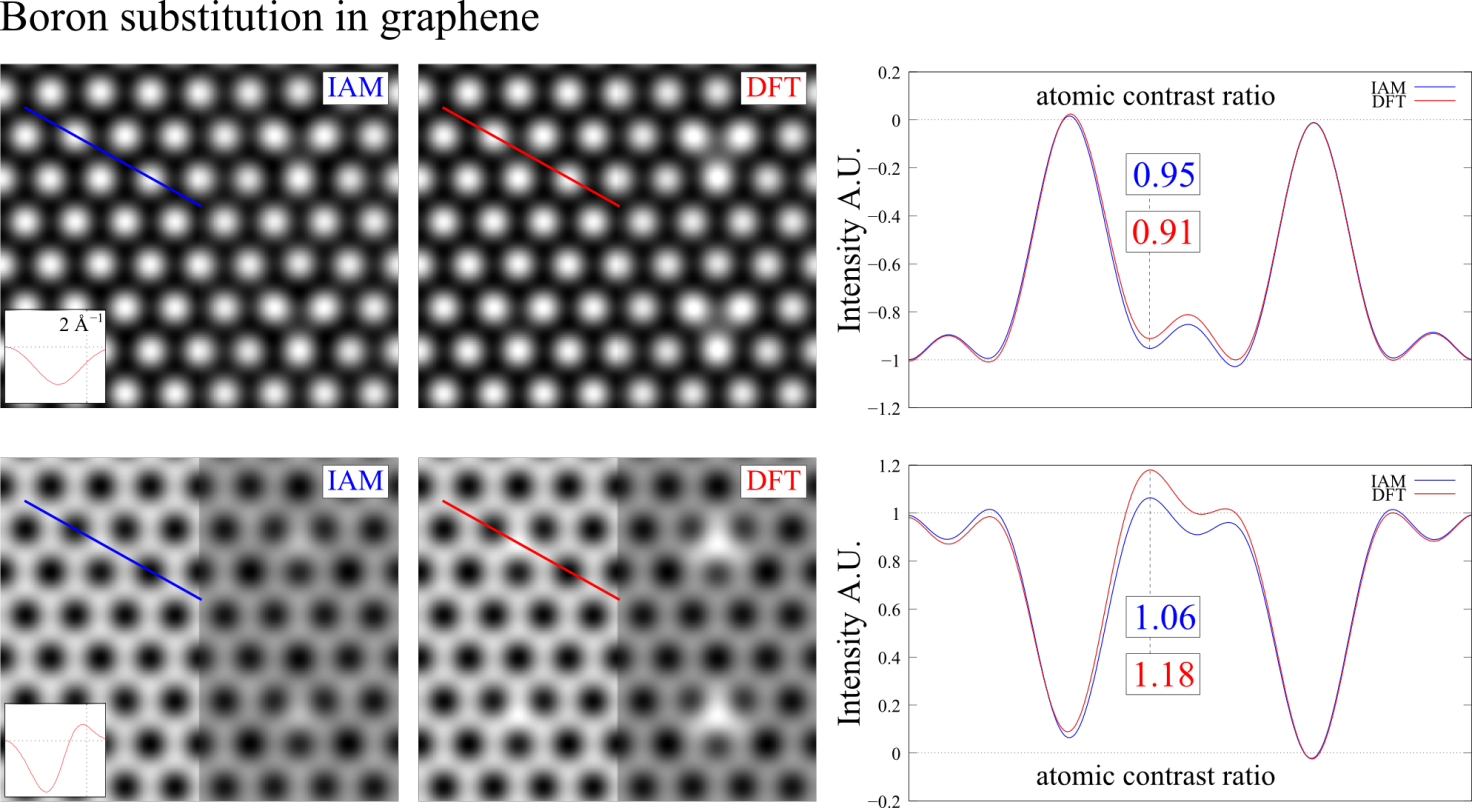
TEM image simulation of boron substitution in graphene for an electron energy of 80 keV. The upper images are for Scherzer defocus *f*_1_ = −9 nm and the lower images are for *f*_2_ = −18 nm. The contrast of the graphene lattice was normalized in all micrographs. This simplifies the comparison between the neutral and the bonded configuration.

The resulting micrographs for the boron substitution are shown in Figure 7. From this we see that the chemical bonding results in weaker contrast of the boron atom. This simplifies the detection of the substitution atom already at Scherzer defocus because the contrast difference between boron and the carbon atoms of the graphene lattice is increased from 5% for neutral atoms (IAM) to 9% in the bonded configuration (DFT). According to the DFT result this difference should be further pronounced by working at higher defocus, which is shown in the lower part of Figure 7.

**Figure 8:**
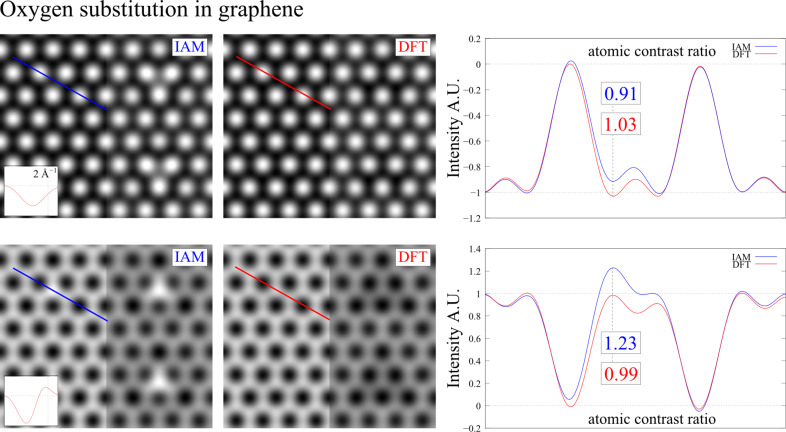
TEM image simulation of oxygen substitution in graphene for the same conditions used in Figure 7.

For the oxygen substitution, shown in Figure 8, we find similar relative contrast changes but reach opposite conclusions, because the polarization of the carbons in the DFT calculation prevents the detection of the oxygen atom in the carbon network, whereas this should be possible according to the IAM result. This may be the reason why we did not detect residual oxygen atoms in reduced graphene oxide [26].

### Oxygen adatom on graphene

The relaxation process resulted in an oxygen atom located at the bridge position between two carbons. The two carbon atoms are bent out of plane by approximately 0.4 Å. The C–C bond is stretched to 1.52 Å and the C–O distance is 1.47 Å, which is in good agreement with previously reported structures [27].

**Figure 9:**
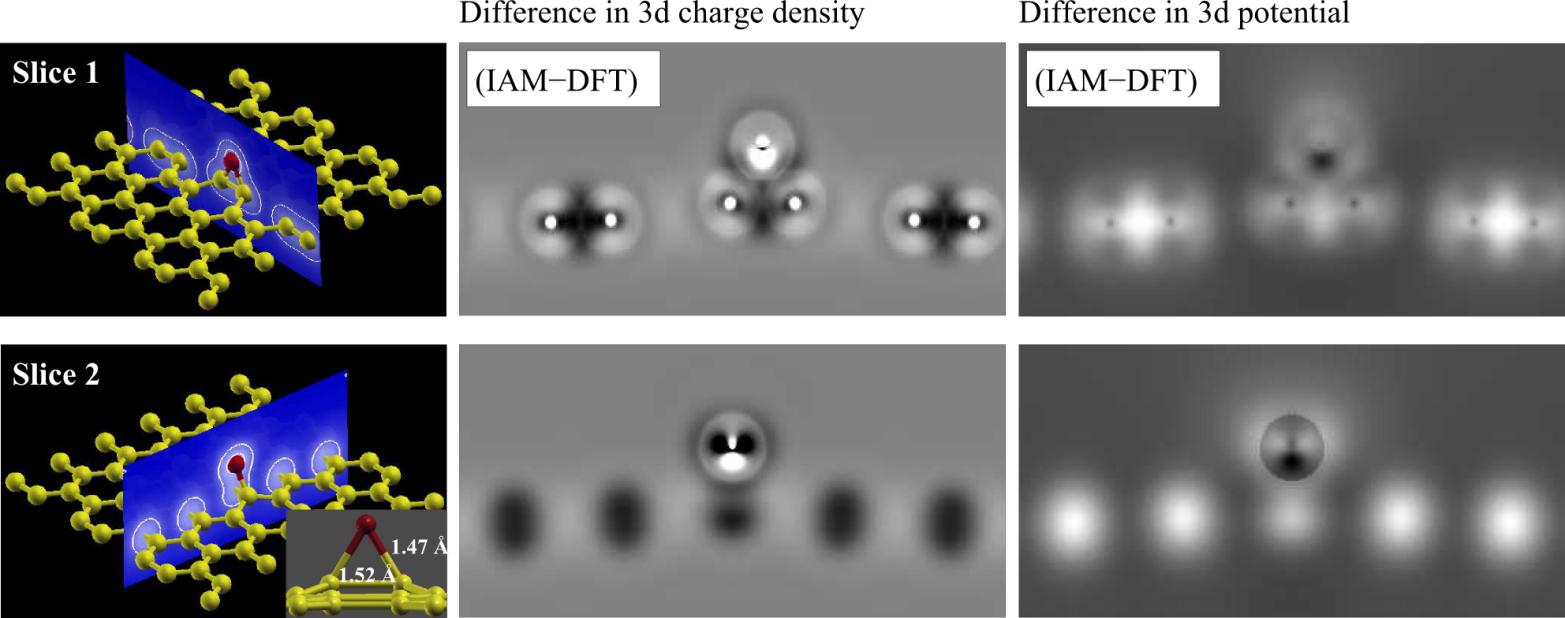
Difference between the 3d electron charge density (center column) and the 3d electrostatic potential (right column) between IAM and DFT for an oxygen adatom on graphene. The top row is for armchair and the bottom row for zig-zag direction. An increase in electron charge density (potential) due to chemical bonding appears as dark (bright) contrast in the difference image.

From analysis of the 3d densities, we found a very interesting charge redistribution in the out-of-plane directions, as can be seen in Figure 9. However, this direction cannot be accessed in a viewing direction orthogonal to the graphene plane.

**Figure 10:**
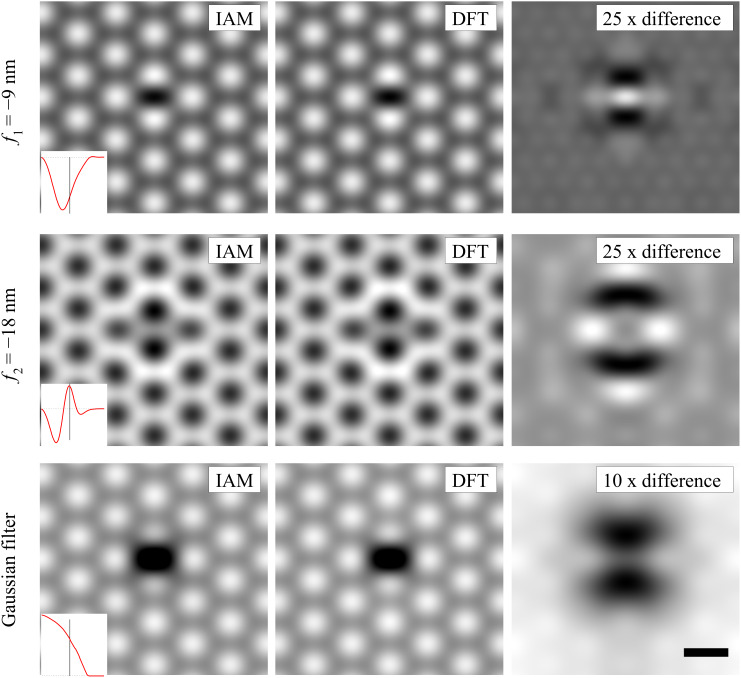
TEM image simulation of an oxygen adatom on graphene for 80 kV. The first row is for Scherzer conditions *f*_1_ = −9 nm and the second row is for higher defocus *f*_2_ = −18 nm. The third row results from applying a low pass filter to the projected potential (1.8 Å Gaussian blur). For each case the inset shows the CTF where the position of the 2.13 Å^−1^ graphene reflex is marked by the black line. For direct comparison, the micrographs, resulting from IAM and DFT, were normalized to the contrast of the graphene lattice and all images within one row are shown on the same grey scale. Therefore the difference images were multiplied by the factor given in the inset. Most interesting, at better transfer of low spatial frequencies, the bonding signal is strongly enhanced. Scale bar is 2.5 Å.

The TEM simulation for normal incidence of the electron beam is shown in Figure 10. Again, the charge transfer around the oxygen atom would be very difficult to detect in a Scherzer defocus image. However, it might be discernable, if the lower spatial frequencies are included in the image. Under the assumption that the oxygen adatom remains stable enough under the electron beam to obtain a sufficient high signal to noise ratio, this might be achievable by applying higher defocus (see second row in Figure 10) or a phase plate (see third row in Figure 10).

## Conclusion

We presented a practical method to include chemical bonding in the HRTEM image simulation process using DFT based scattering potentials recently applied in [13]. Hence, an all-electron calculation was set up based on a previously relaxed atomic configuration. As we have shown the WIEN2k software is well suited for this task as the initial potential is in good agreement with commonly used scattering potentials and the subsequent iteration process acts as a relatively minor perturbation. The potential itself is not very sensitive to the DFT input parameters. However, as the electrostatic potential is divergent at the position of the atomic nuclei, care has to be taken during the discretization process. We found that a sampling rate of 30 ppÅ in combination with a cutoff method produced reasonably accurate results. This 3d potential can subsequently be used for multislice TEM simulation, however this was not necessary for the single layer materials studied here. The influence of chemical bonding can be analyzed by comparing the IAM charge density, corresponding potential and TEM image with those obtained from the DFT calculation.

This analysis was demonstrated for the substitutions of a single boron and a single oxygen atom in graphene as well as for an oxygen adatom on graphene. The relative changes are very similar to the ones we found previously for the single atom nitrogen substitution [13], where we were able to validate the advantage of the DFT calculation over the isolated atom model experimentally. For the oxygen substitution we find exactly the same situation as for the nitrogen defect: The electron charge density in the area surrounding the substitution is decreased due to polarization of the neighboring carbons, resulting in weaker shielding of the core potential and increased TEM contrast of the substitution atom. For boron the situation is exactly the inverse. However, the implication on the final TEM image is not very intuitive because it depends on the absolute potential values: For nitrogen, the increase of the contrast due to charging enables the detection whereas for oxygen this increase disables the detection. On the contrary, the decrease of the boron contrast simplifies the detection because the relative contrast difference to the carbon lattice is increased from 5% in the neutral to 9% in the bonded configuration, where only the latter is significantly above the experimental accuracy of 3% [13].

We conclude that chemical bonding must be included in comparative HRTEM image simulations whenever very small signals are analyzed. Here we want to emphasize that the key requirement for this kind of analysis is the well defined specimen geometry rather than the single layer thickness of the model systems used in this study. Earlier calculations showed that, whenever a high enough experimental accuracy is achieved, bonding effects should be detectable for a wide variety of materials [2,8]. This offers the possibility to gain experimental insight into the electronic charge distribution of the specimen at the atomic scale by HRTEM.

## Supporting Information

File 1 WIEN2k convergence tests for ideal graphene

File 2 Program to get 3D WIEN2k potentials (Phyton script, rename to .py).

## References

[1] Gemming T, Möbus G, Exner M, Ernst F, Rühle M (1998). J Microsc.

[2] Deng B, Marks L D (2006). Acta Crystallogr, Sect A.

[3] Kirkland E J (1998). Advanced Computing in Electron Microscopy.

[4] Hébert C (2007). Micron.

[R5] 5Hofer, W. A. *A Guide to simulation of STM images and spectra from first principles*: bSKAN 3.6; www.liv.ac.uk/~whofer/stm/bskan_guide.pdf, 2005.

[6] Rose H (1990). Optik.

[7] Haider M, Rose H, Uhlemann S, Kabius B, Urban K J (1998). Electron Microsc.

[8] Deng B, Marks L D, Rondinelli J M (2007). Ultramicroscopy.

[9] Zhu Y, Wu L, Tafto J (2003). Microsc Microanal.

[10] Zuo J M, Spence J C H, O’Keeffe M (1988). Phys Rev Lett.

[11] Ciston J, Kim J, Haigh S, Kirkland A, Marks L (2010). Ultramicroscopy.

[12] Meyer J C, Chuvilin A, Algara-Siller G, Biskupek J, Kaiser U (2009). Nano Lett.

[13] Meyer J C, Kurasch S, Park H J, Skakalova V, Künzel D, Groß A, Chuvilin A, Algara-Siller G, Roth S, Iwasaki T (2011). Nat Mater.

[14] O’Keefe M A (1992). Ultramicroscopy.

[15] Thust A (2009). Phys Rev Lett.

[16] Lehmann M, Lichte H (2002). Microsc Microanal.

[17] Doyle P A, Turner P S (1968). Acta Crystallogr, Sect A.

[18] Coulthard M A (1967). Proc Phys Soc, London.

[19] Kresse G, Furthmüller J (1996). Phys Rev B.

[20] Blaha P, Schwarz K, Madsen G K H (2001). An Augmented Plane Wave + Local Orbitals Program for Calculating Crystal Properties.

[21] Arai M wien2venus.py.

[22] Cottenier S (2002). Density Functional Theory, the family of (L)APW-methods: a step-by-step introduction.

[23] Perdew J, Burke K, Ernzerhof M (1996). Phys Rev Lett.

[24] Schowalter M, Lamoen D, Rosenauer A, Kruse P, Gerthsen D (2004). Appl Phys Lett.

[25] Hÿtch M J, Stobbs W M (1994). Ultramicroscopy.

[26] Gómez-Navarro C, Meyer J C, Sundaram R S, Chuvilin A, Kurasch S, Burghard M, Kern K, Kaiser U (2010). Nano Lett.

[27] Yan J-A, Chou M Y (2010). Phys Rev B.

