# Supplementary material for: Simulation of bonding effects in HRTEM images of light element materials
Source: Beilstein J Nanotechnol. 2011 Jul 19;2:394–404. doi: 10.3762/bjnano.2.45 (PMC3190611; doi:10.3762/bjnano.2.45)
Supplement: File 1 — WIEN2k convergence tests for ideal graphene [file Beilstein_J_Nanotechnol-02-394-s001.pdf]

Supporting Information

for

Simulation of bonding effects in HRTEM images of light element materials

Simon Kurasch*[1], Jannik C. Meyer[1,2], Daniela Künzel[3], Axel Groß[3] and Ute Kaiser[1]

Address: [1]Central Facility for Electron Microscopy, Group of Electron Microscopy of Materials Science, University of Ulm, 89081 Ulm, Germany; [2]New address: University of Vienna, Department of Physics, 1090 Vienna, Austria and [3]Institute of Theoretical Chemistry, University of Ulm, 89069 Ulm, Germany

Email: Simon Kurasch – simon.kurasch@uni-ulm.de

* Corresponding author

WIEN2k convergence tests for ideal graphene

The WIEN2k potential calculation comes with some new input parameters, compared to an IAM simulation. In order to set up meaningful DFT calculations, these parameters need to be chosen carefully and, for new material systems, convergence tests are unavoidable.

K-points

The first parameter we tested was the number of k-points that is needed. Therefore a number of DFT calculations of ideal graphene were set up using an increasing number of k-points. The basis size was kept constant (RKMAX = 7). The most important quantity to determine a good k-mesh is the convergence of the total energy. From Figure 1 it can be seen that the computer time per cycle scales linearly with the number of k-points, as it should be, and that the total energy is converged already for 100k.

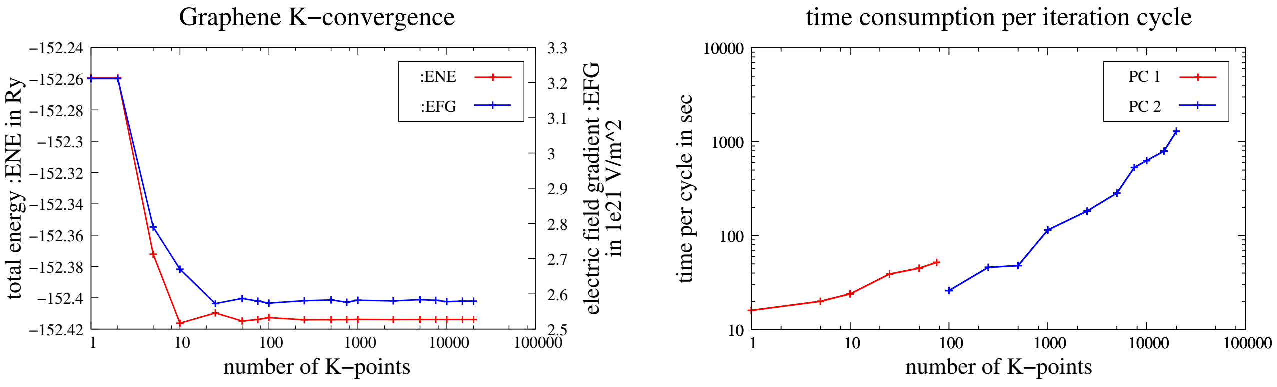

Figure 1: Graphene k convergence test. On the left we see the dependence of the total energy and the electric field gradient on the number of k-points. On the right the corresponding computation time is visualized.

As we are mainly interested in the projected potential we also checked how this quantity is influenced by the number of k-points. The projected potential was calculated from a single slice parallel to the beam direction. Each 2d slice was normalized to the smallest value and no cutoff was used during the projection process. Four calculations (50k, 100k, 1000k and 10000k) were compared to the calculation with the highest number of k-points that was performed (20000k). The range of the absolute difference (left side of Figure 2) for each calculation is relatively small while the absolute high of the difference plot is probably very sensitive to the normalization. On the right side of Figure 2 we see the relative difference between the projected potentials V_z obtained for difference numbers of k-points compared to the 20000k calculation in % that was obtained by: $100 \cdot (V_z - V_z^{20000k})/V_z^{20000k}$. Surprisingly, the projected potential does not converge smoothly with the number of k-points: The 100k calculation is in much better accordance to the 20000k calculation than the 1000k calculation. One reason might be the low DFT convergence conditions that were used for these calculations (charge convergence: -cc 0.0001 C and energy convergence: -ee 0.0001 Ry).

In conclusion it can be said that 100k are enough and that the projected potential is not very sensitive to the number of k-points. As the calculation time scales only linear with the number of k-points this parameter is not critical for the quantity we are interested in.

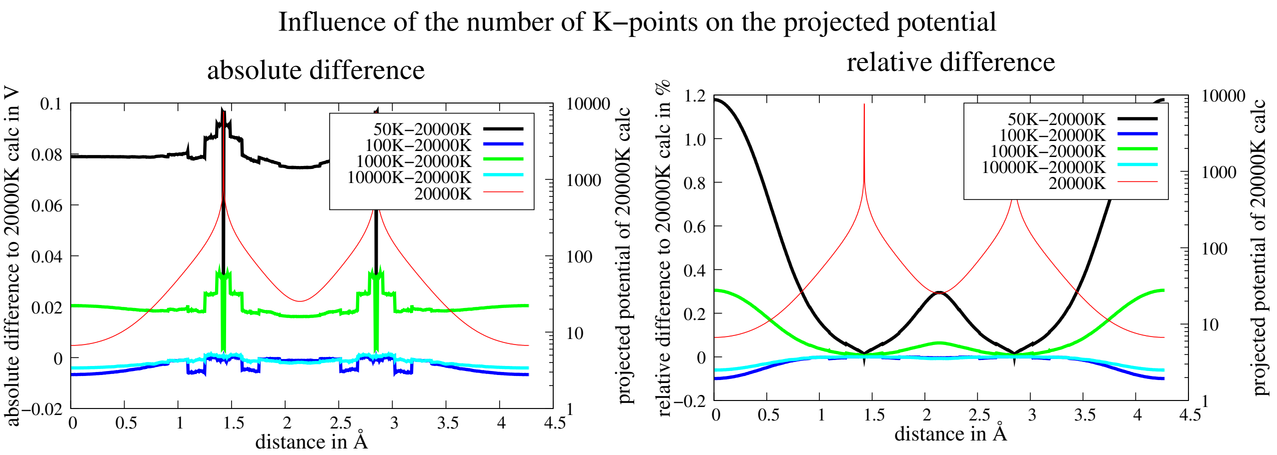

Figure 2: Dependence of the projected potential on the number of k-points used during the DFT calculation. The red line corresponds to the projected potential of the 20000k calculation.

Size of the basis set

The second parameter we tested was the size of the basis set that is determined by the RKMAX value. This value was increased starting from 5.5 to 9.0 with a step width of 0.5. In contrast to the k-convergence test the DFT convergence conditions were increased to: -cc 0.00001 C and -ec 0.00001 Ry.

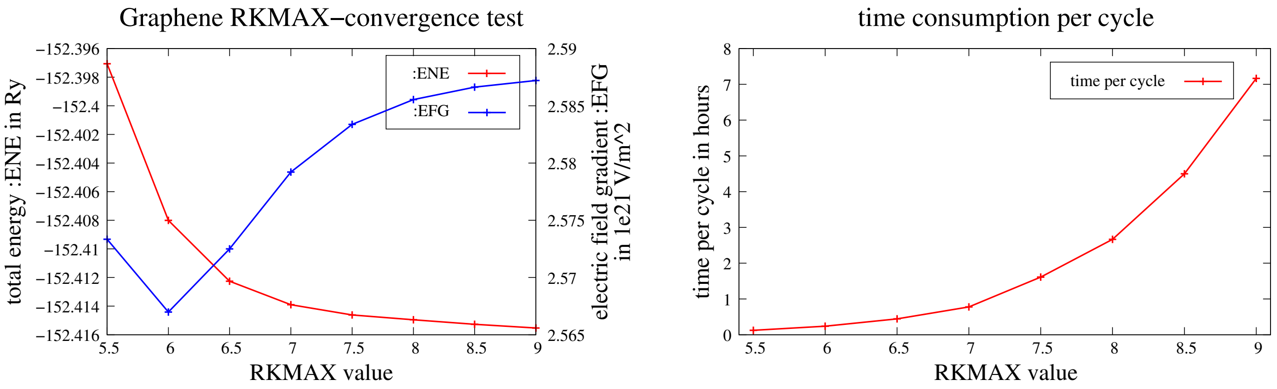

Figure 3: Graphene RKMAX convergence test. On the left we see the dependence of the total energy and the electric field gradient on the size of the basis set (determined by RKMAX value) used during the DFT calculation. On the right hand side we see that high values of RKMAX are very expensive from the computational point of view.

From Figure 3 we can learn that it is necessary to use a very high value of RKMAX when making DFT calculations of graphene. The WIEN2k default value is RKMAX = 7.0 where in this study it

should be necessary to use a value of RKMAX = 9.0. For bigger systems this high value may not be affordable due to strongly increasing computation time. We again calculated the projected potential for several RKMAX values and compared them to the calculation with the highest accuracy. The results are visualized in Figure 4 and we see that luckily the projected potential is not very sensitive to RKMAX: The relative difference between RKMAX = 7 and RKMAX = 9 (green line on the right side of Figure 4) is only 0.06% at its maximum.

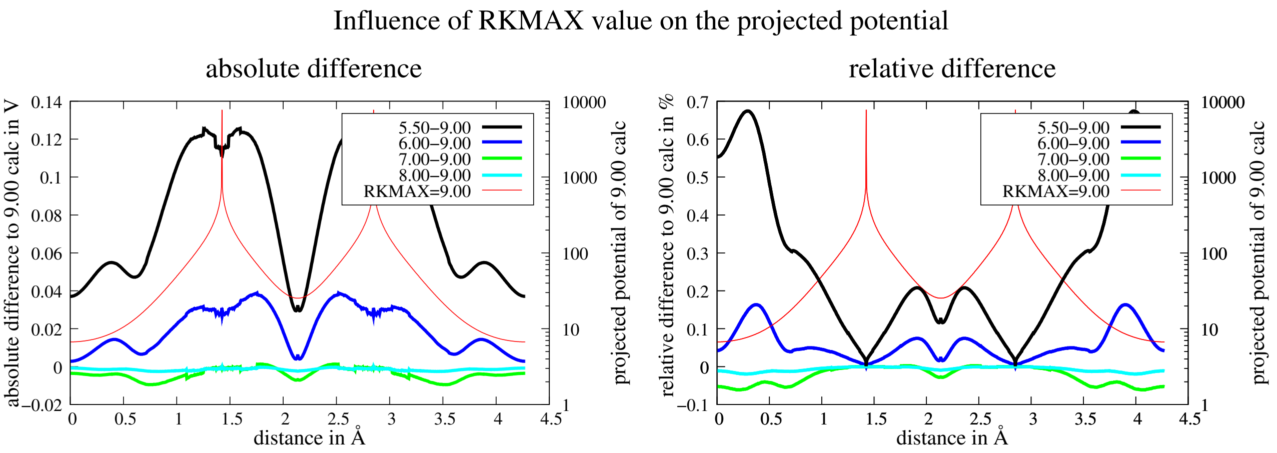

Figure 4: Influence of the basis set size used in the DFT calculation on the projected potential of graphene. The red line corresponds to the projected potential of the RKMAX = 9 calculation.

For ideal graphene it is of course possible to use RKMAX = 9 whereas for bigger systems calculated with RKMAX = 7 the error in the projected potential due to the too small basis set should be relatively small.

Fourier expansion of charge density

The accuracy of the Fourier expansion of the charge density is determined by GMAX where the default value is 12. We performed DFT calculations for several GMAX values ranging from 10 to 20 with a step width of 2. All calculations were performed with RKMAX = 5, k-mesh of $19 \times 19 \times 1$ (500k) and fine convergence conditions of -cc 0.00001 C and -ec 0.00001 Ry.

From Figure 5 we see that it should be better to increase GMAX to 16 instead of using the default value of 12.

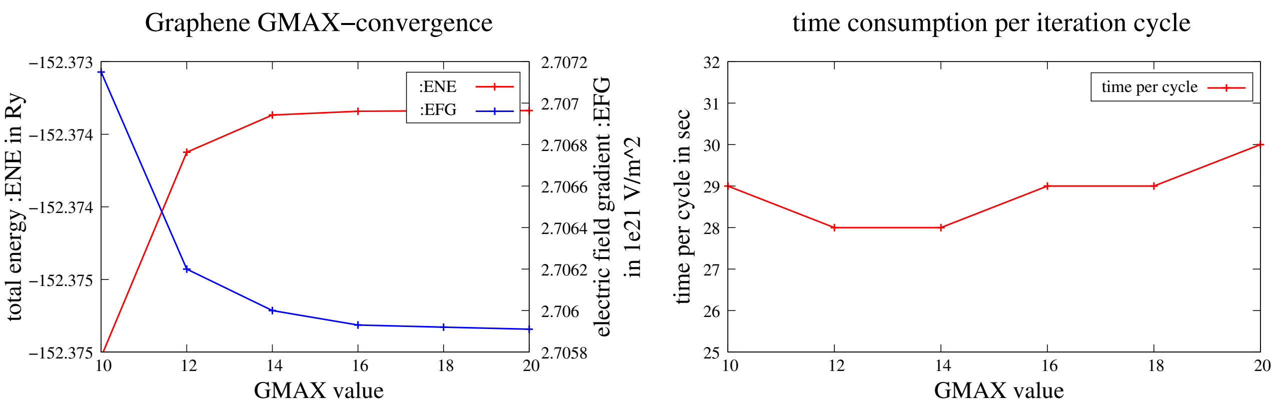

Figure 5: Graphene GMAX convergence test. On the left we see the dependence of the total energy and the electric field gradient on the accuracy of the Fourier expansion used during the DFT calculation. On the right hand side we see that the DFT calculation time does not depend on the GMAX value.

Furthermore, from Figure 5, it is possible to see that the calculation time of the SCF cycle does not depend on the GMAX value. On the other hand the charge density and potential files are becoming bigger when GMAX is increased as there are more Fourier coefficients. This influences the time needed to slice these files what is visualized in Figure 6 where the time consumed to create one slice is printed against the GMAX value.

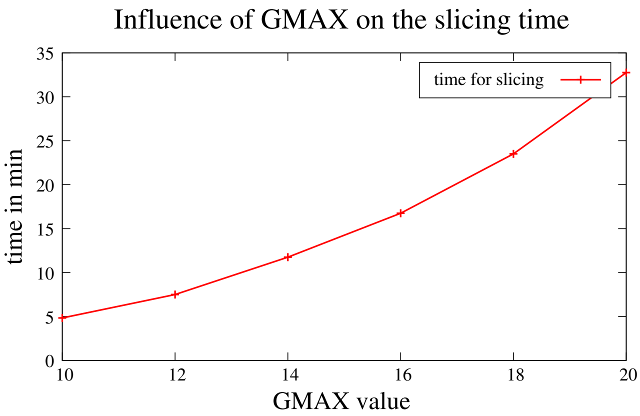

Figure 6: Correlation between the GMAX value and the time to create one 2d potential slice.

We also checked the influence of the GMAX parameter on the projected potential and found that for GMAX = 10 the relative error is up to 4% while for the default value of 12 the error is smaller than 0.25%.

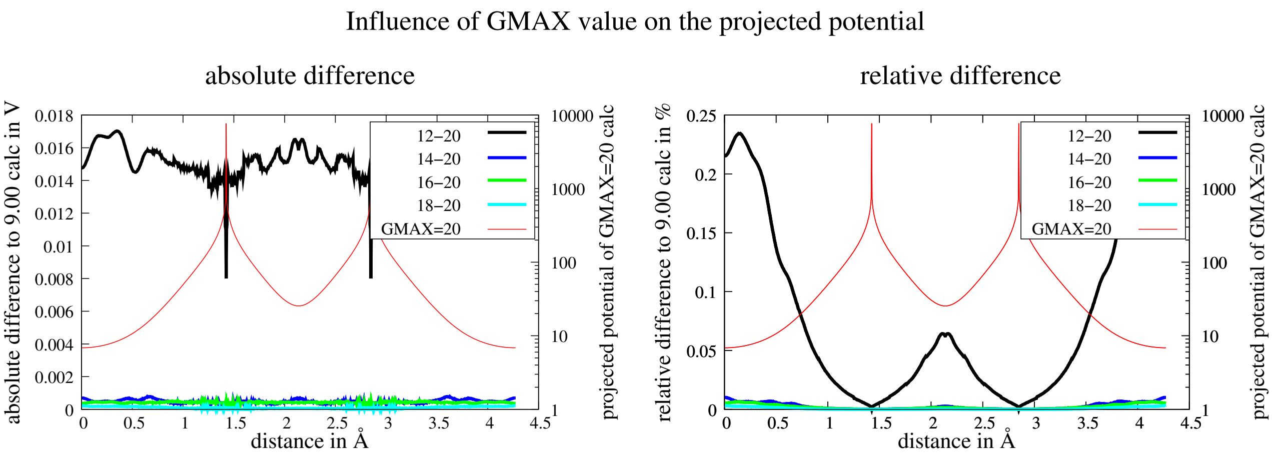

Figure 7: Influence of the Fourier expansion on the projected potential of graphene. The red line corresponds to the projected potential of the GMAX = 20 calculation.

Layer separation

Graphene is a true 2d material in 3d space while our DFT calculations were always using 3d unit cells and 3d periodic boundary conditions. How large do we have to make the unit cell in z-direction to 'isolate' the graphene layers? The graphene layer separation was increased from 5 Å to 35 Å with a stepsize of 5 Å and the total energy and electric field gradient were plotted against the layer separation in Figure 8. One problem that complicates the interpretation of this study is the fact that each calculation was using a different k-grid. We always were using 500k for the WIEN2k input but the software constructed different k-grids with variating number of k-points for each case so the results are not directly comparable. Nevertheless, it appears that a vacuum separation of $20-25$ Å is sufficient to suppress the interaction between two neighboring graphene layers.

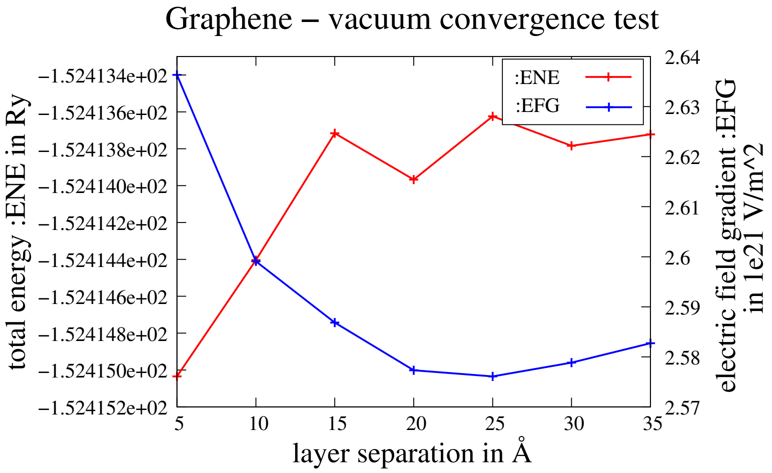

Figure 8: DFT calculations for graphene using different layer separations.
